# Supplementary material for: Discovery of Hydrazineyl Amide Derivative of Pseudolaric Acid B for Reprogramming Tumor-Associated Macrophages Against Tumor Growth
Source: Molecules. 2025 May 8;30(10):2088. doi: 10.3390/molecules30102088 (PMC12114375; doi:10.3390/molecules30102088)
Supplement: Supplementary file 1 [file molecules-30-02088-s001.zip › molecules-3530088-supplementary.pdf]

Discovery of Hydrazineyl Amide Derivative of Pseudolaric Acid B for Reprogramming Tumor-Associated Macrophages Against Tumor Growth

Xia Peng[1,2,3,†], Siqi Yu[4,5,†], Lin Xu[4,5,†], Qinghua Wang[4], Lin Yang[4,5], Yi Su[1,2,3], Zhirou Xiong[2,3], Mengjie Shao[4], Meiyu Geng[1,2,3,6], Ao Zhang[4], Lei Zhang[7,*], Jing Ai[2,3,6*], Chunyong Ding[4,5,*]

[1] School of Chinese Materia Medica, Nanjing University of Chinese Medicine, Nanjing 210023, China

[2] State Key Laboratory of Drug Research, Shanghai Institute of Materia Medica, Chinese Academy of Sciences, Shanghai 201203, China

[3] School of pharmacy, University of Chinese Academy of Sciences, Beijing 100049, China

[4] Shanghai Frontiers Science Center of Drug Target Identification and Delivery, National Key Laboratory of Innovative Immunotherapy, School of Pharmaceutical Sciences, Shanghai Jiao Tong University, Shanghai 200240, China

[5] School of Pharmaceutical Sciences, Zunyi Medical University, Zunyi 563000, China

[6] Shandong Laboratory of Yantai Drug Discovery, Bohai Rim Advanced Research Institute for Drug Discovery, Yantai, Shandong 264117, China

[7] Key Laboratory for Modernization of Qiandongnan Miao & Dong Medicine, Qiandongnan Traditional Medicine Research & Development Center, School of Life and Health Science, Kaili University, Kaili 556011, China

[†]These authors contributed equally to this work.

*Corresponding authors:

Lei Zhang, Tel: (86) 855-8558300, Fax: (86) 855-8553376, E-mail: lzhang0412@163.com;

Jing Ai, Tel: (86) 21- 6807788-6295, Fax: (86) 21- 68077888, E-mail: jai@simm.ac.cn
Chunyong Ding, Tel: (86) 21-34204016, Fax: (86) 21-34204016, E-mail: chunding@sjtu.edu.cn

Contents

1. **Figure S1-27**……………………………………………………….………S4-19

2. **Table S1**………………………………………………………………… S20

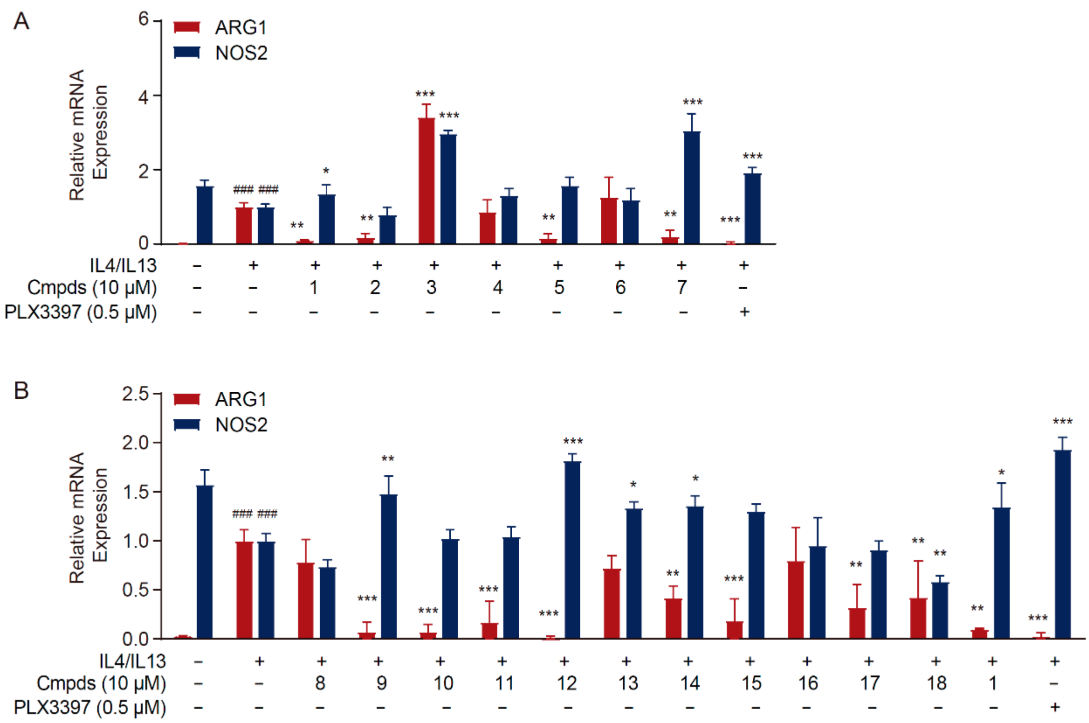

Figure S1. The effects of compounds on protumor phenotype of macrophages. Quantitative RT-PCR analysis for mRNA levels of ARG1 and NOS2 in RAW 264.7 cells treated with IL-4/IL-13 alone or combined with indicated compounds (10 μM) for 12 h. Cells treated with IL-4/IL-13 (20 ng/mL) were used as the stimulated controls and cells without treatment as the non-stimulated control. The data shown are representative results from three biological replicates of two independent experiments. Data was represented as 2-ΔΔCt in the form of means ± SD. #P < 0.05, ##P < 0.01, ###P < 0.001 vs non-stimulated control group, *P < 0.05, **P < 0.01, ***P < 0.001 vs stimulated control group. P values were determined by one way ANOVA.

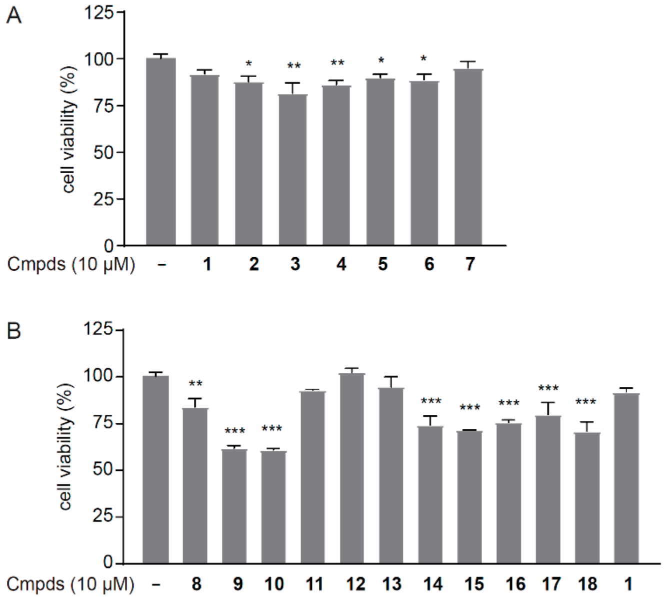

Figure S2. Effects of natural pseudolaric acids **1-4**, **6** and their O-pyranosides **5**, **7** as well as the derivatives **8-18** of **1** on Raw264.7 cell viability. Raw264.7 cells were treated with IL-4/IL-13 alone or combined with indicated compounds (10 μM) for 12 h. After incubation, the cell viability was determined using a cell counting kit (CCK-8, Dojindo, Japan) assay.

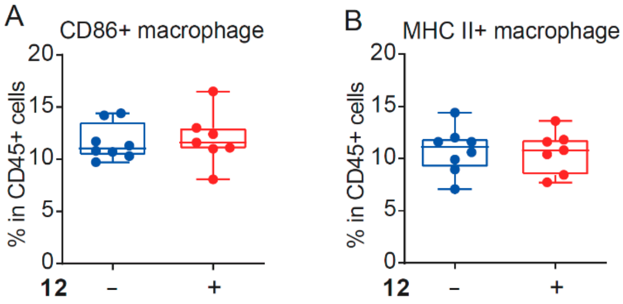

Figure S3. Flow cytometric analysis of immune subsets in the Hepa1-6 tumor model treated with vehicle or Compound 12. Tumor tissues collected 2 h after the last treatment with Compound 12. The infiltration of $CD86^+$ TAMs (A) and MHC II^+ TAMs (B) were tested (n = 8 mice in vehicle group and n = 7 mice in 25 mg/kg 12 treated group).

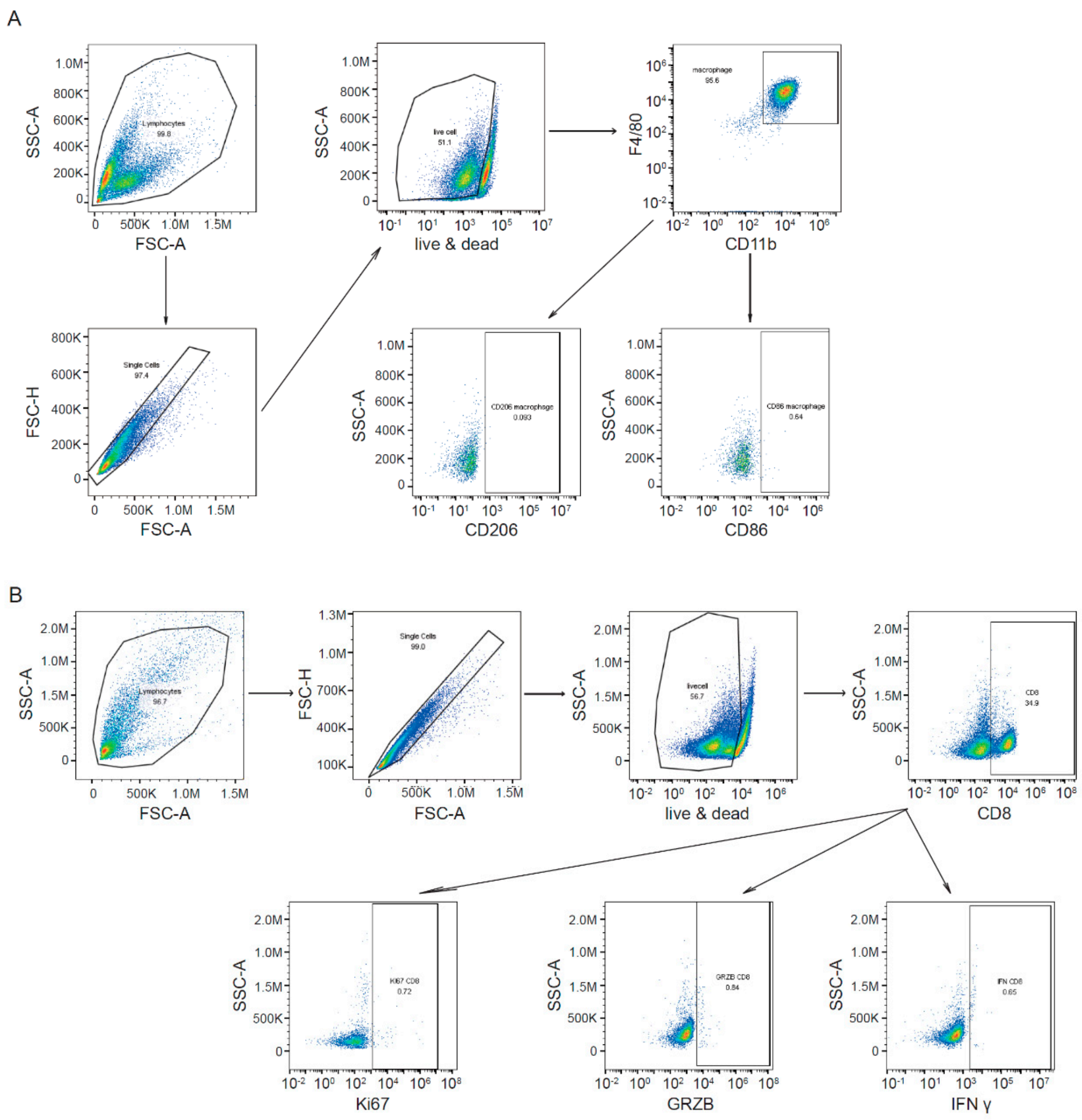

Figure S4. The gating strategies for Macrophage (A), CD8+T cell (B) in flow cytometry analyses.

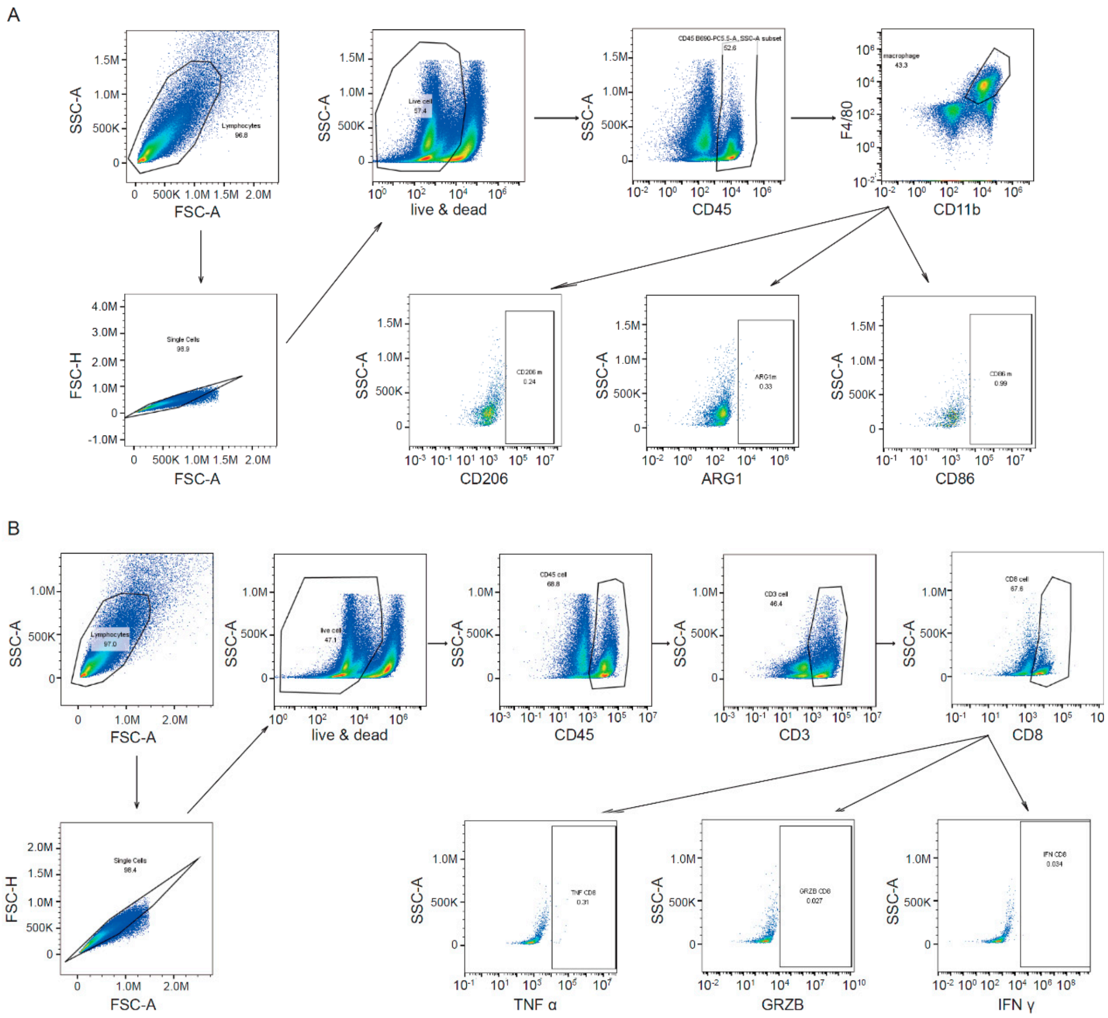

Figure S5. The gating strategies for Macrophage (A), CD8+T cell (B) in flow cytometry analyses of hepa1-6 tumor-infiltrating immune cells.

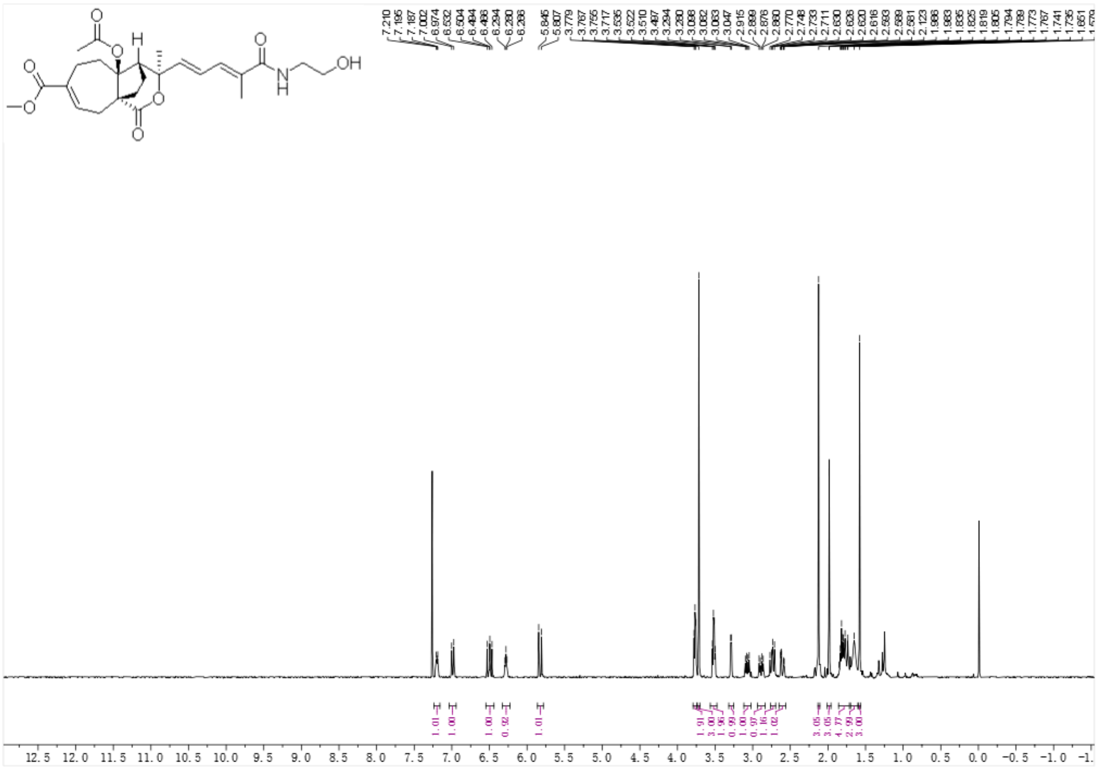

Figure S6. ^{1}H NMR spectrum of **8** (400 MHz, $CDCl_3$)

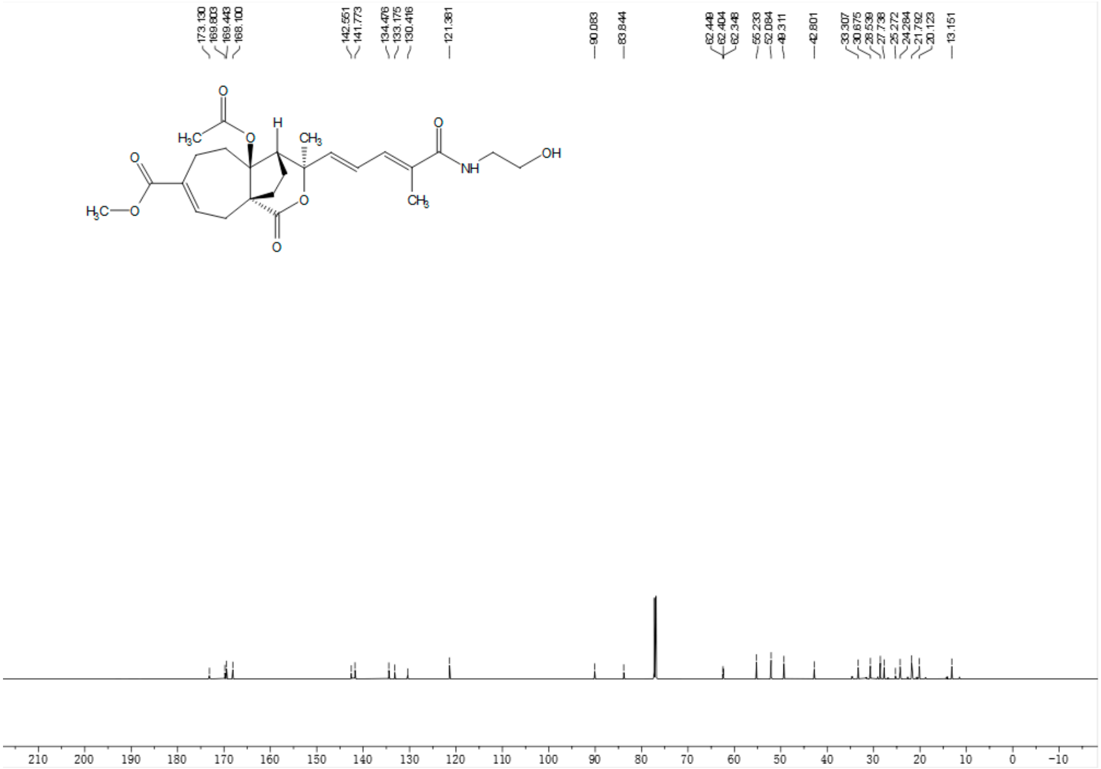

Figure S7. ^{13}C NMR spectrum of **8** (700 MHz, $CDCl_3$)

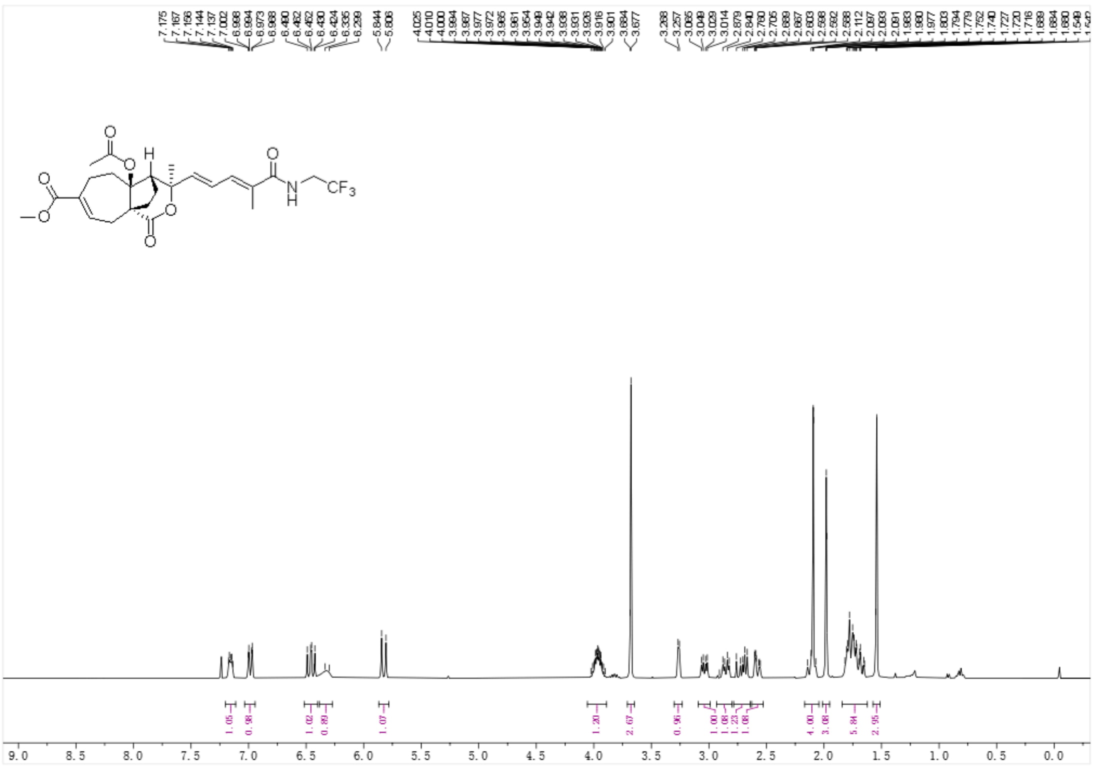

Figure S8. ^{1}H NMR spectrum of **9** (400 MHz, $CDCl_3$)

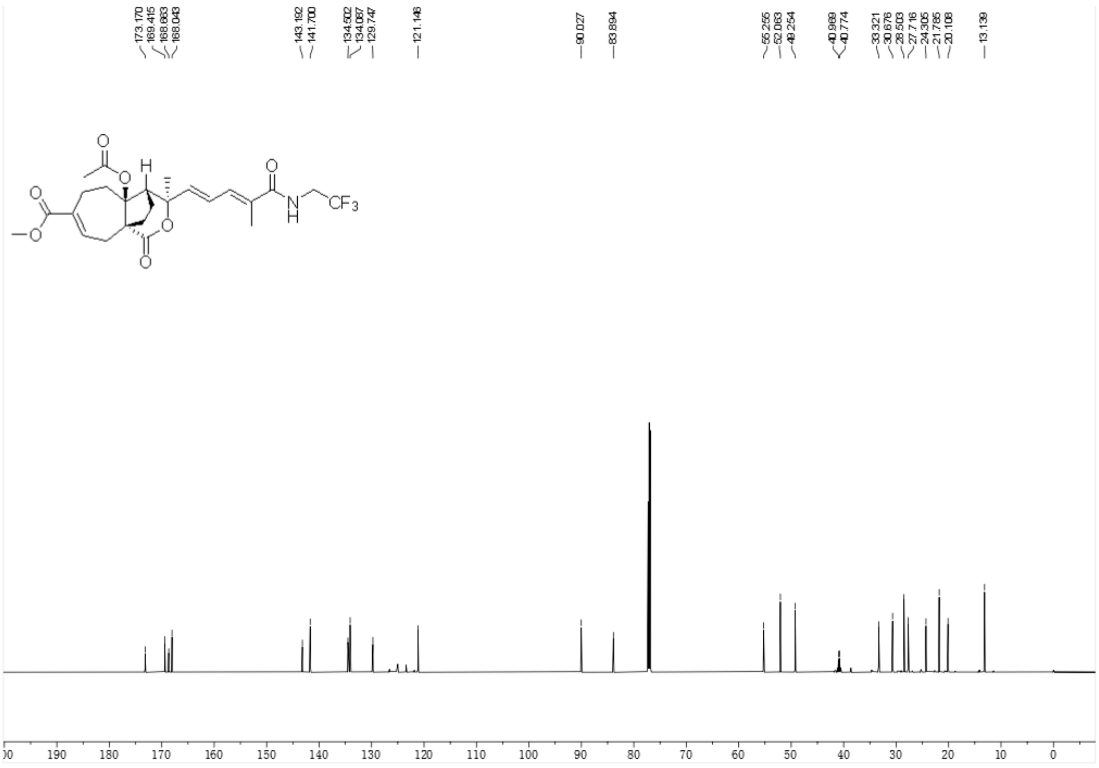

Figure S9. ^{13}C NMR spectrum of **9** (700 MHz, $CDCl_3$)

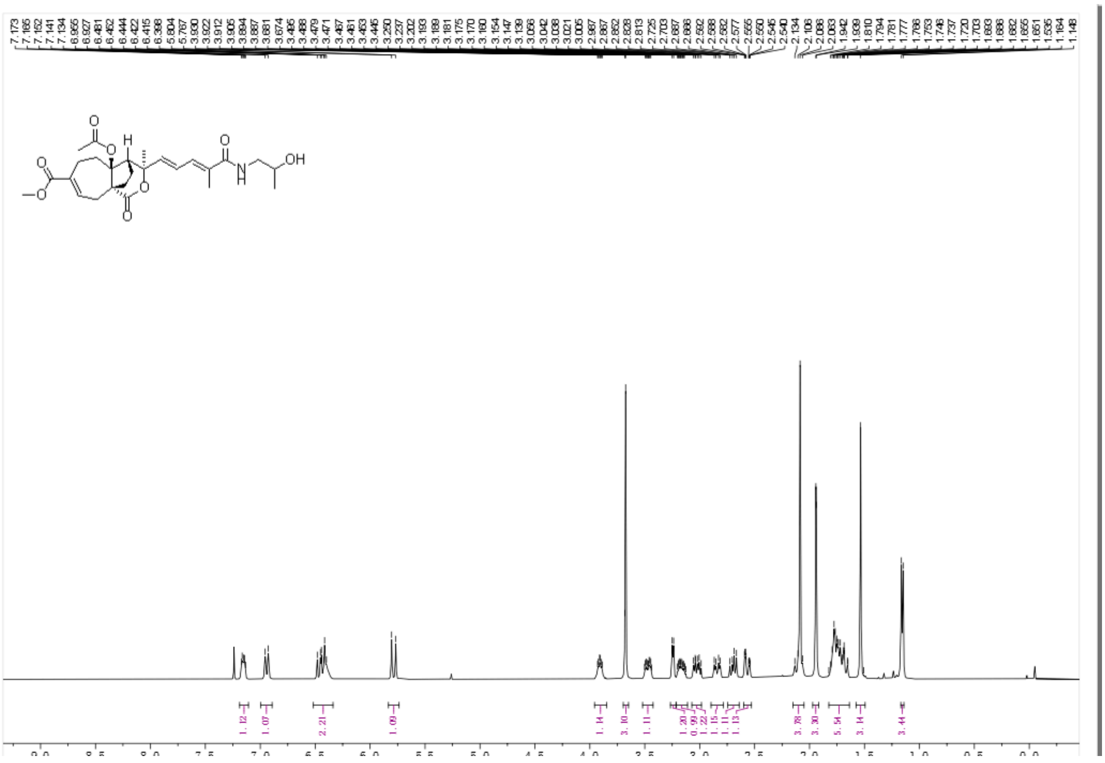

Figure S10. ^{1}H NMR spectrum of **10** (400 MHz, $CDCl_3$)

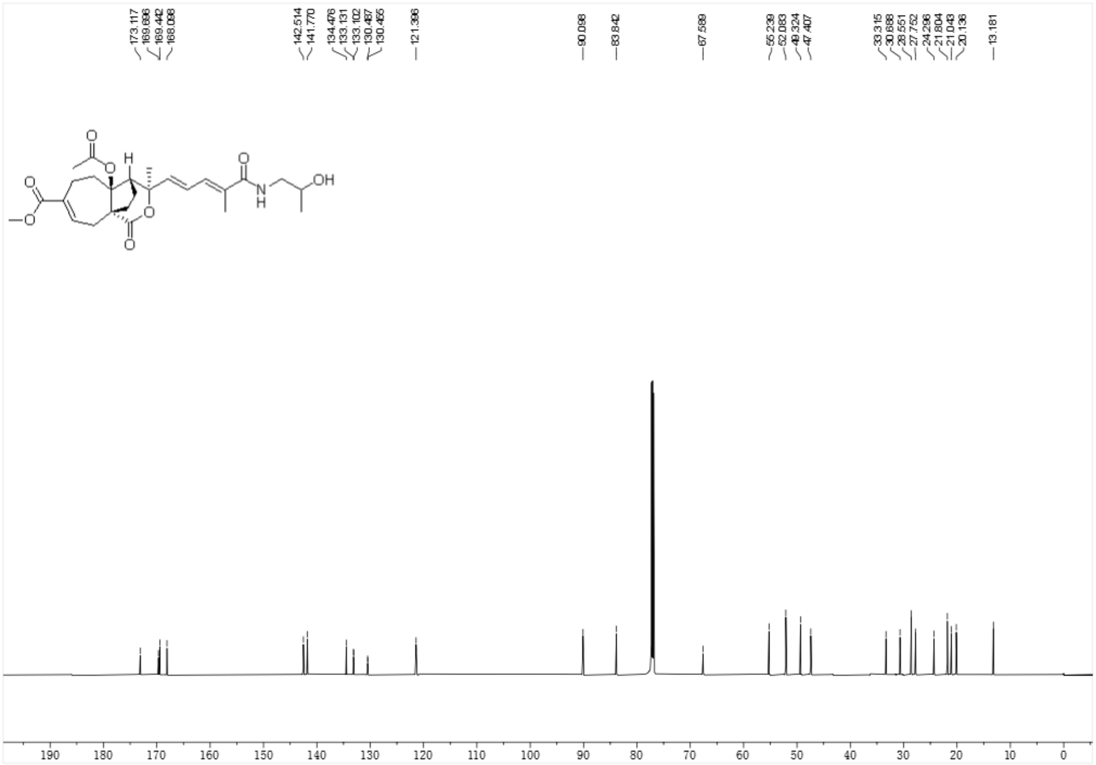

Figure S11. ^{13}C NMR spectrum of **10** (700 MHz, $CDCl_3$)

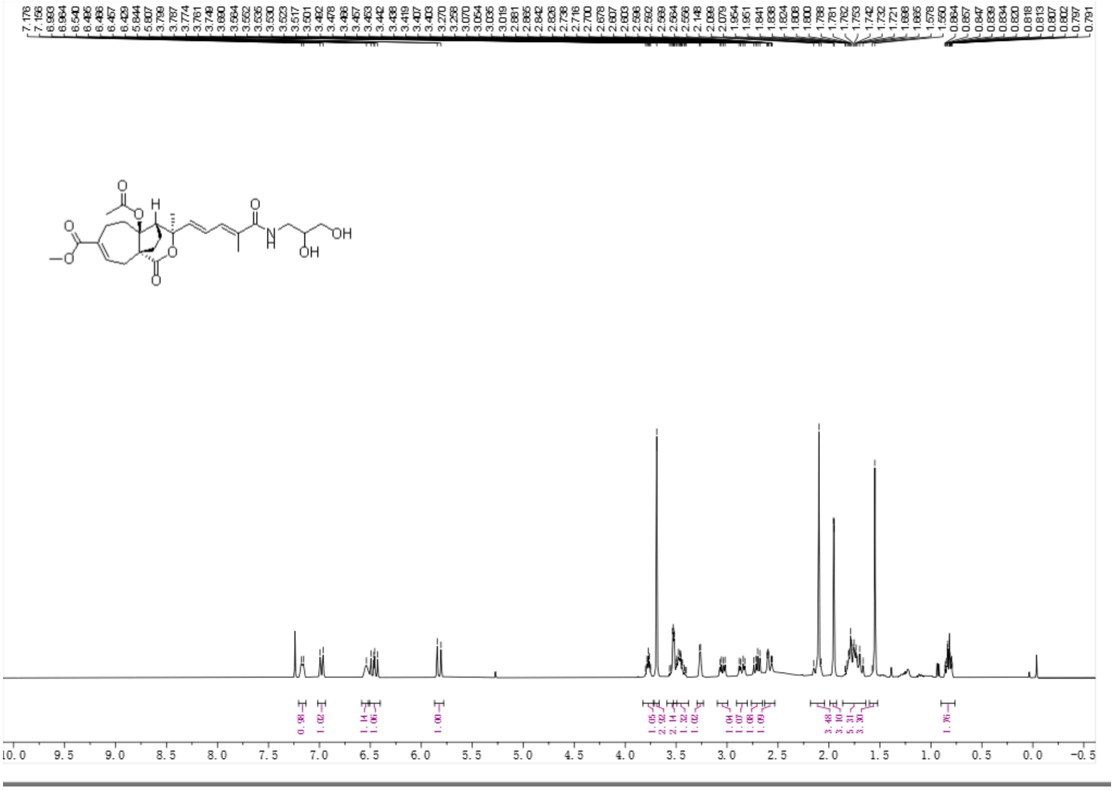

Figure S12. ^{1}H NMR spectrum of **11** (400 MHz, $CDCl_3$)

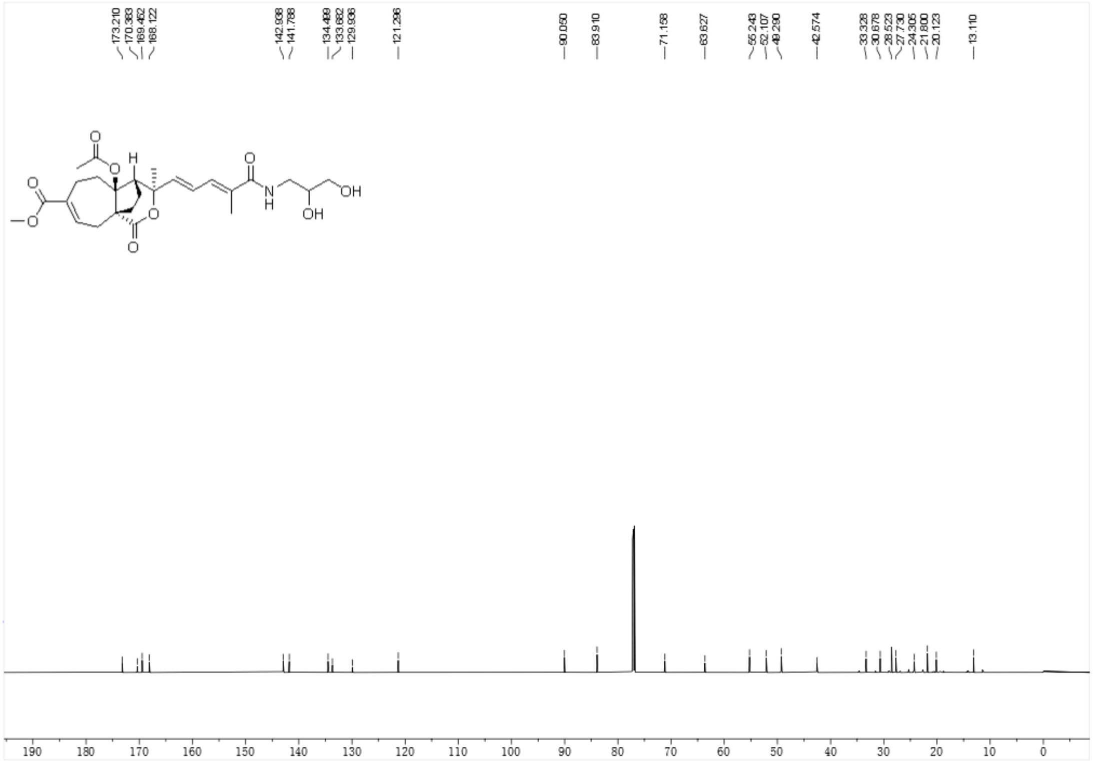

Figure S13. ^{13}C NMR spectrum of **11** (700 MHz, $CDCl_3$)

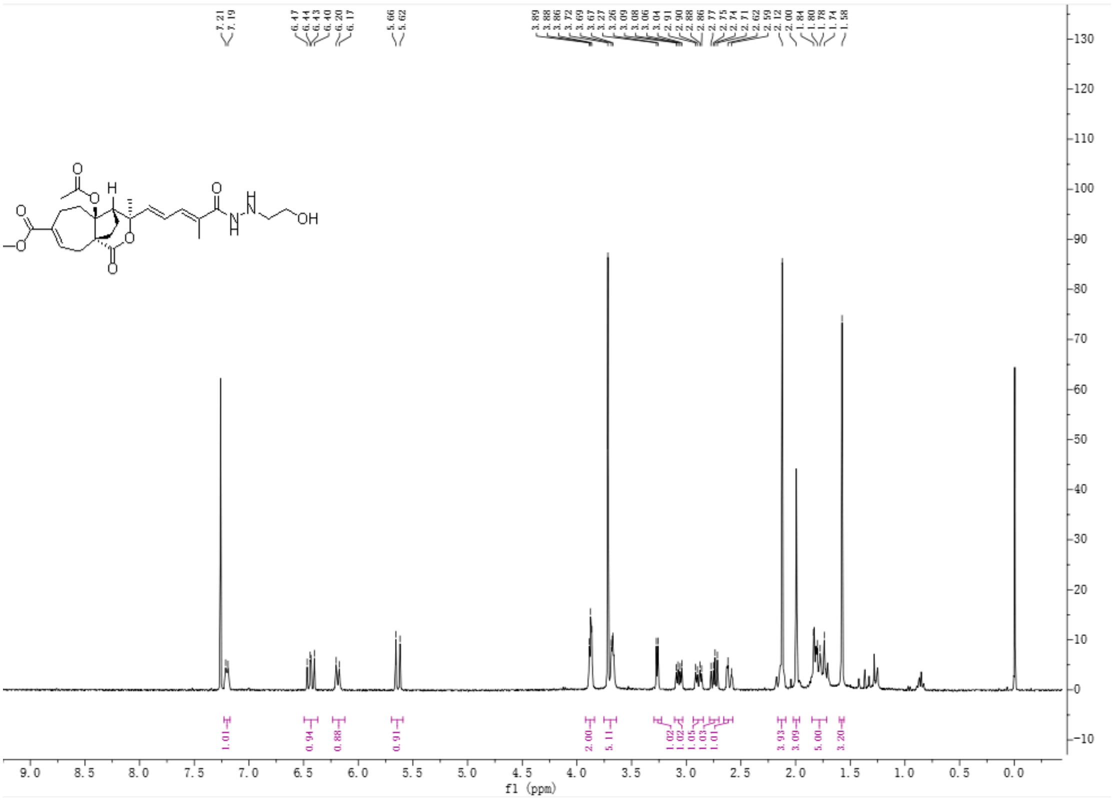

Figure S14. ^{1}H NMR spectrum of **12** (400 MHz, $CDCl_3$)

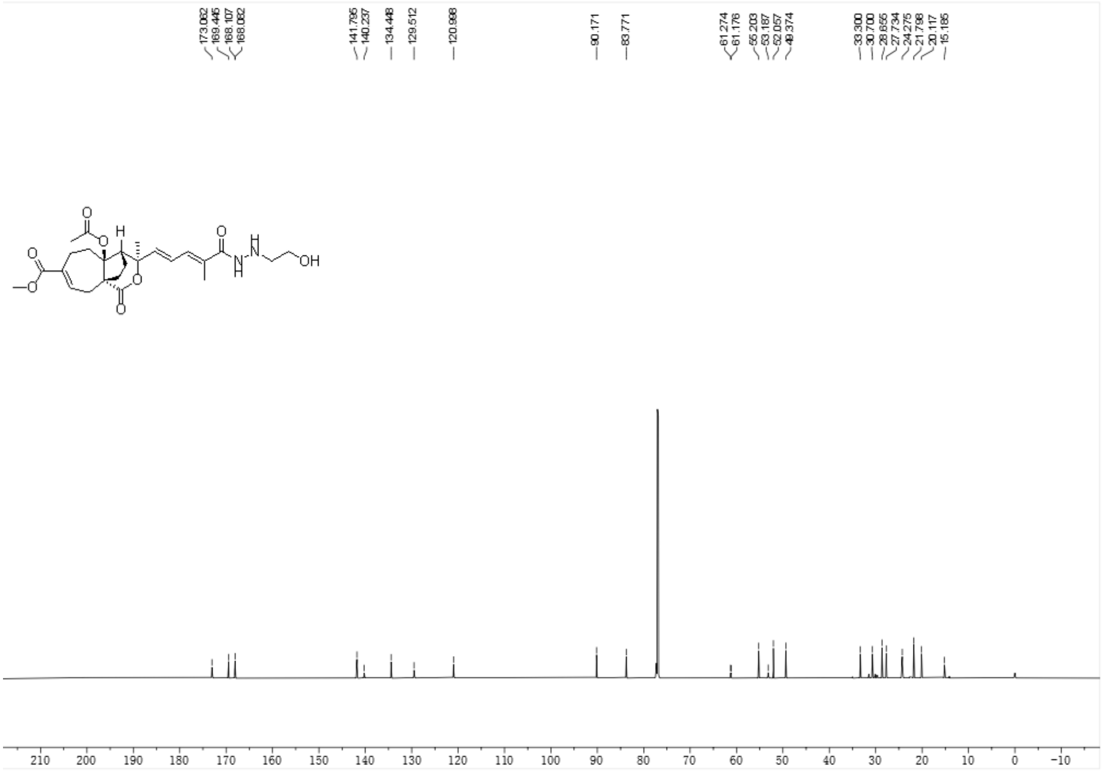

Figure S15. ^{13}C NMR spectrum of **12** (700 MHz, $CDCl_3$)

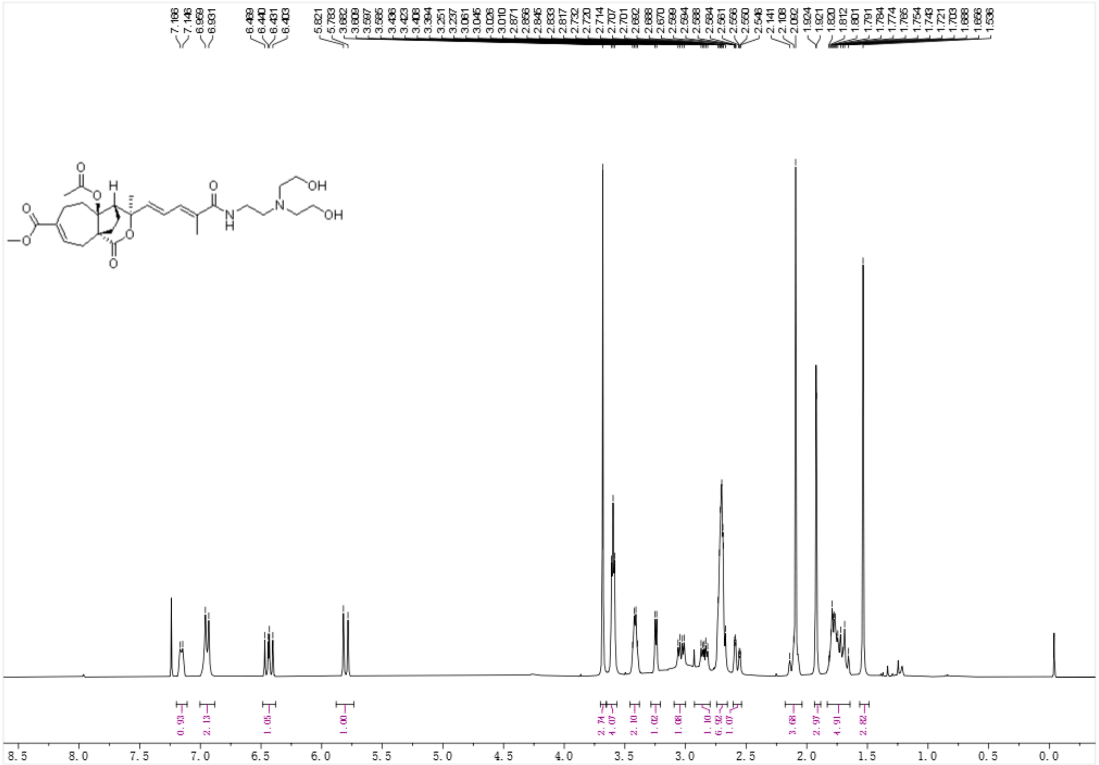

Figure S16. ^{1}H NMR spectrum of **13** (400 MHz, $CDCl_3$)

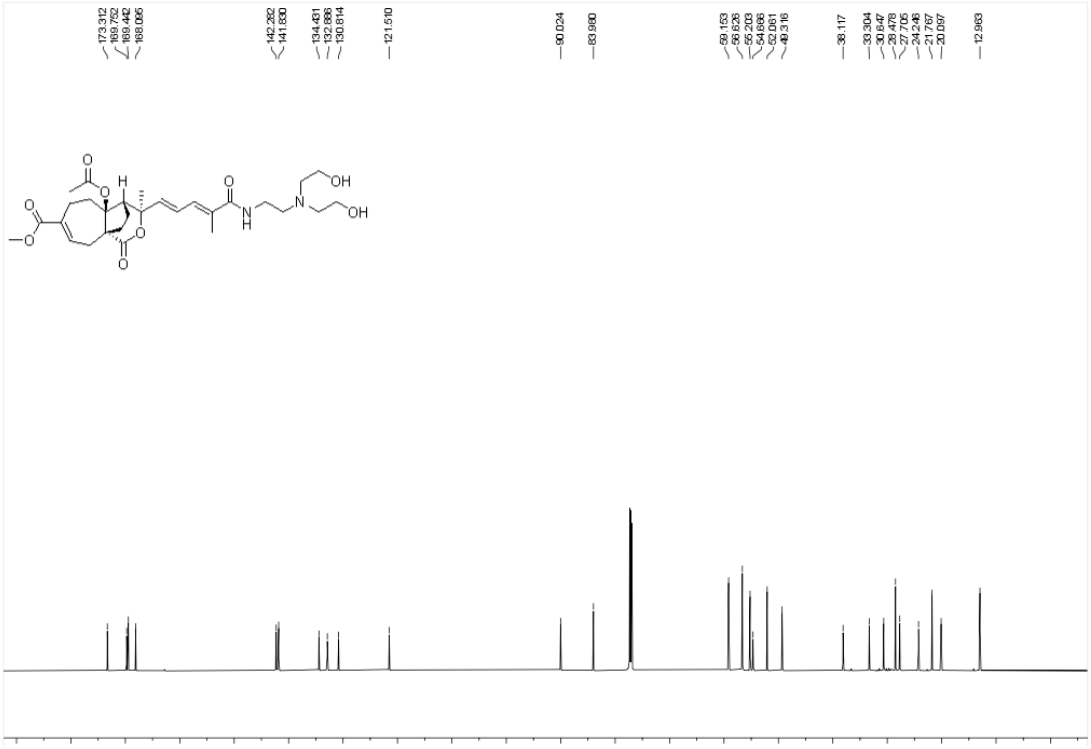

Figure S17. ^{13}C NMR spectrum of **13** (700 MHz, $CDCl_3$)

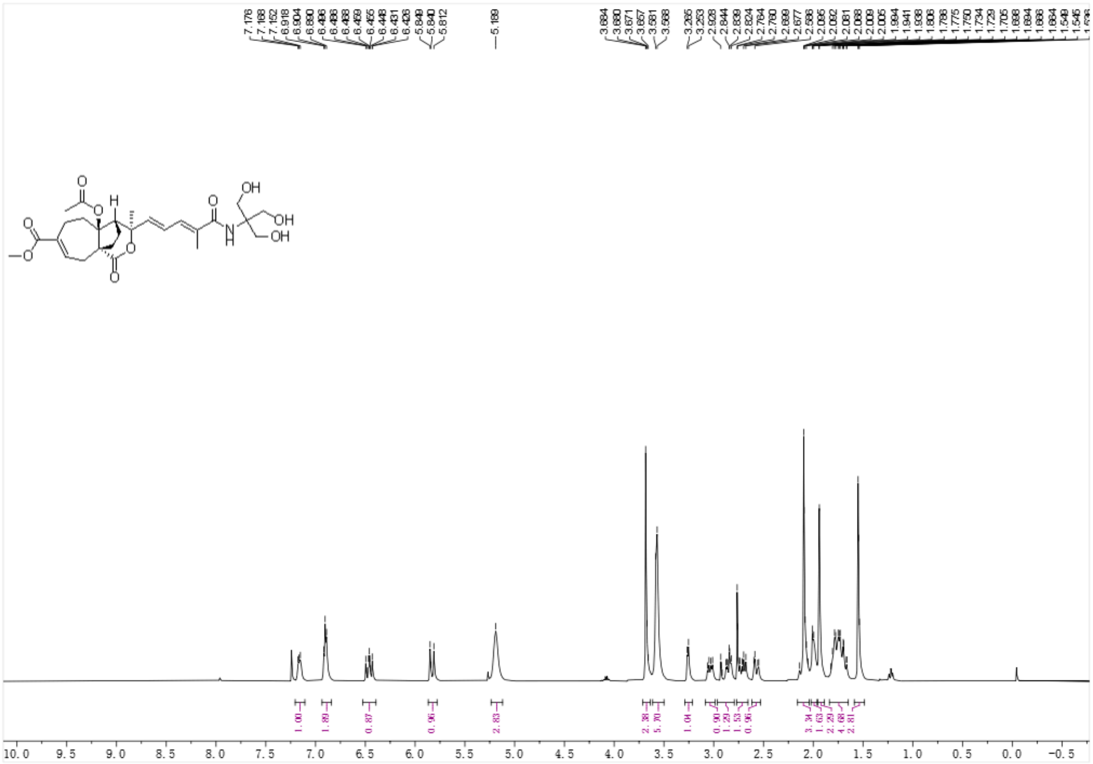

Figure S18. ^{1}H NMR spectrum of **14** (400 MHz, $CDCl_3$)

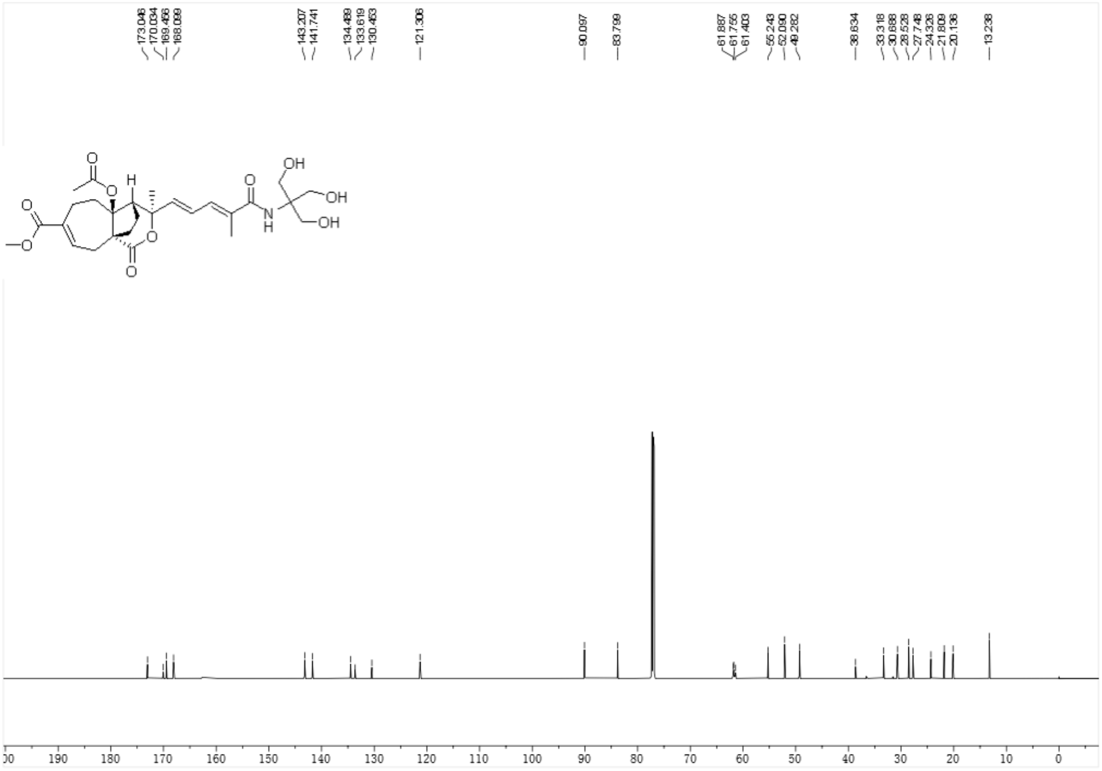

Figure S19. ^{13}C NMR spectrum of **14** (700 MHz, $CDCl_3$)

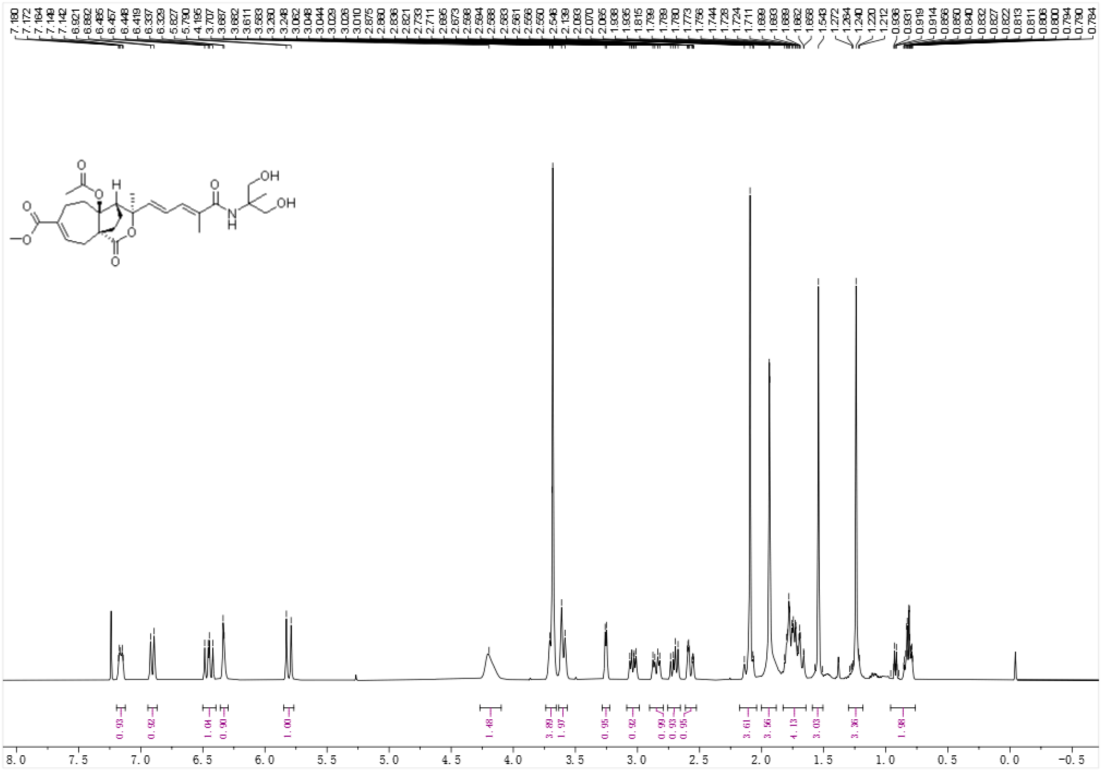

Figure S20. ^{1}H NMR spectrum of **15** (400 MHz, $CDCl_3$)

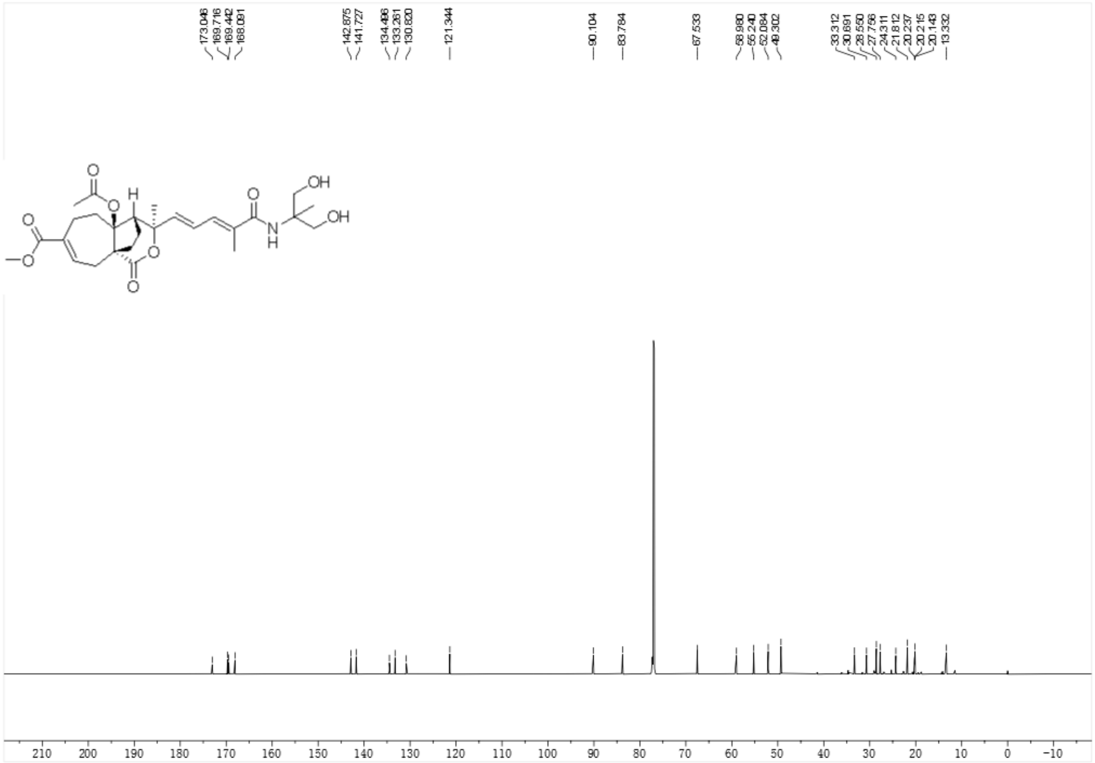

Figure S21. ^{13}C NMR spectrum of **15** (700 MHz, $CDCl_3$)

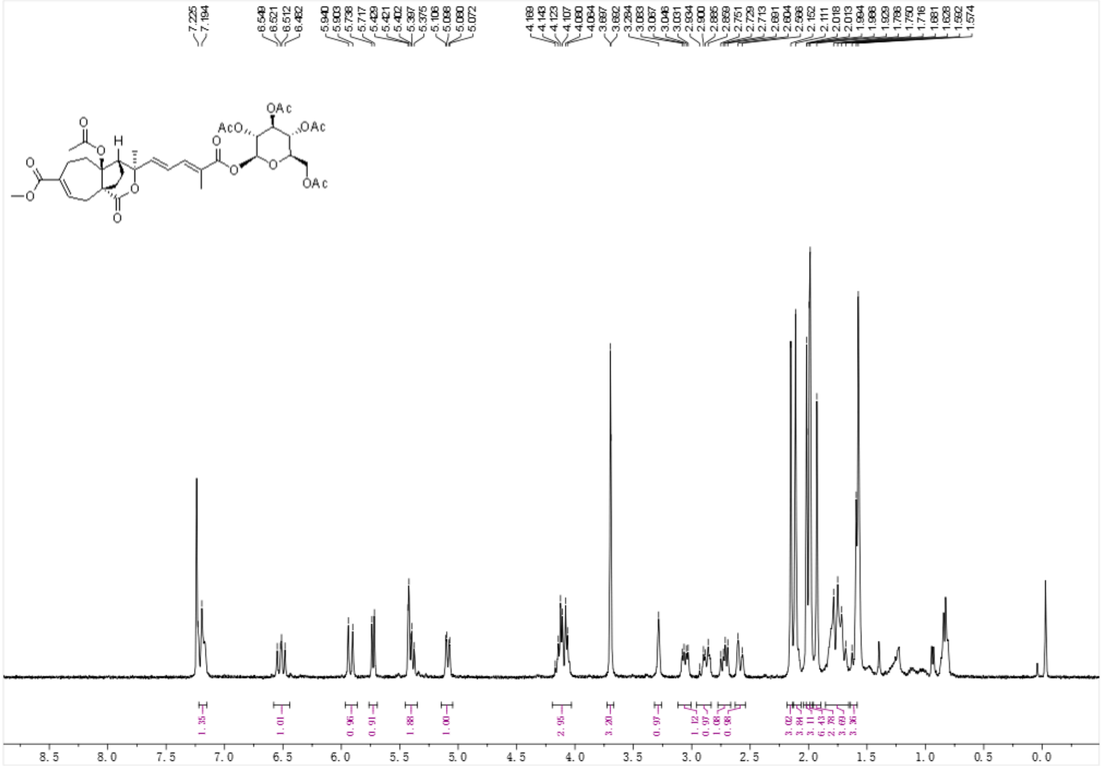

Figure S22. ^{1}H NMR spectrum of **16** (400 MHz, $CDCl_3$)

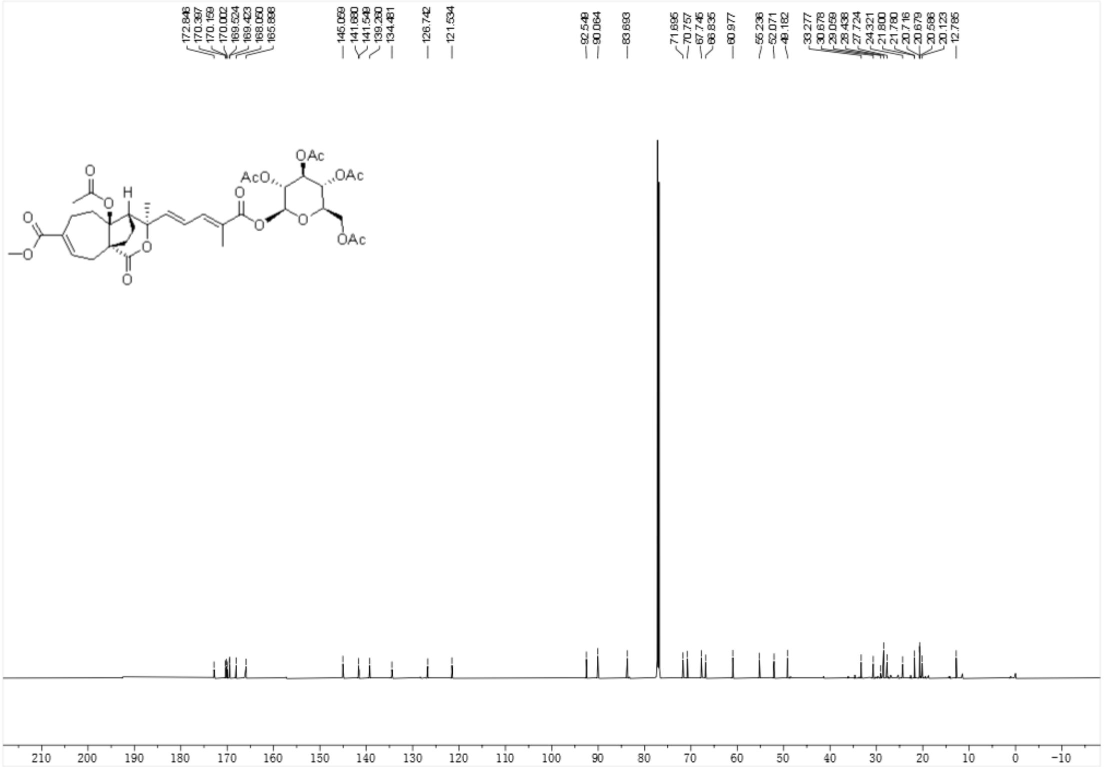

Figure S23. ^{13}C NMR spectrum of **16** (700 MHz, $CDCl_3$)

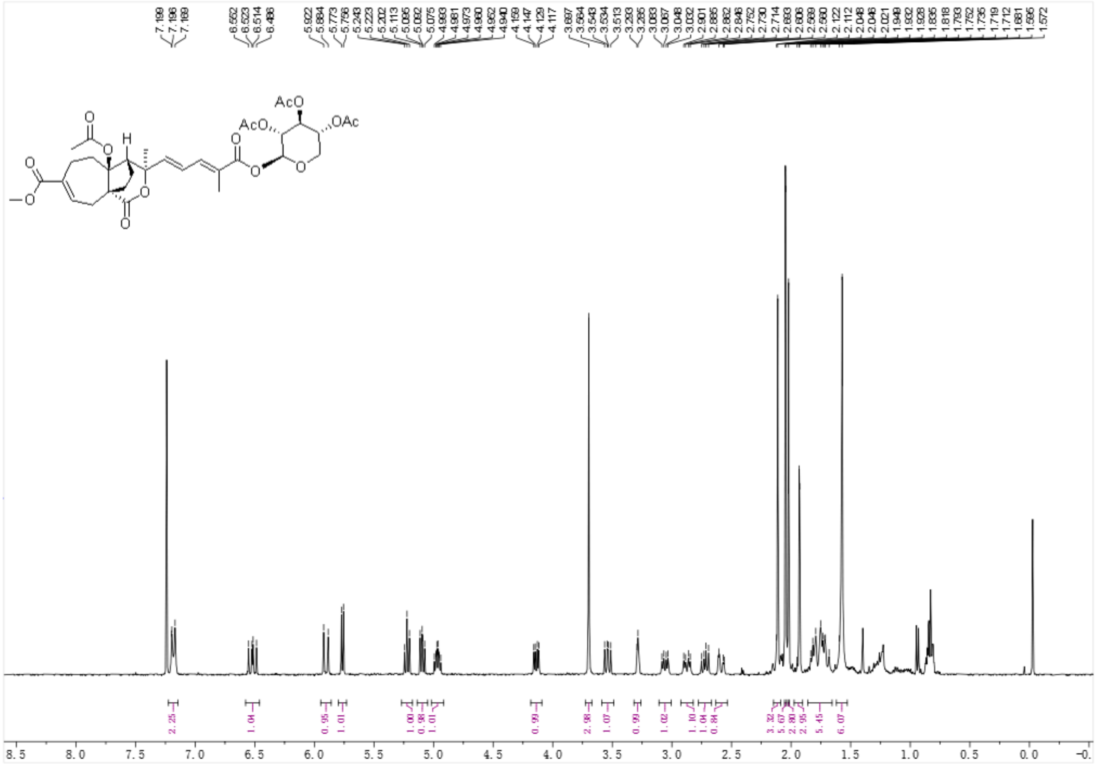

Figure S24. ^{1}H NMR spectrum of **17** (400 MHz, $CDCl_3$)

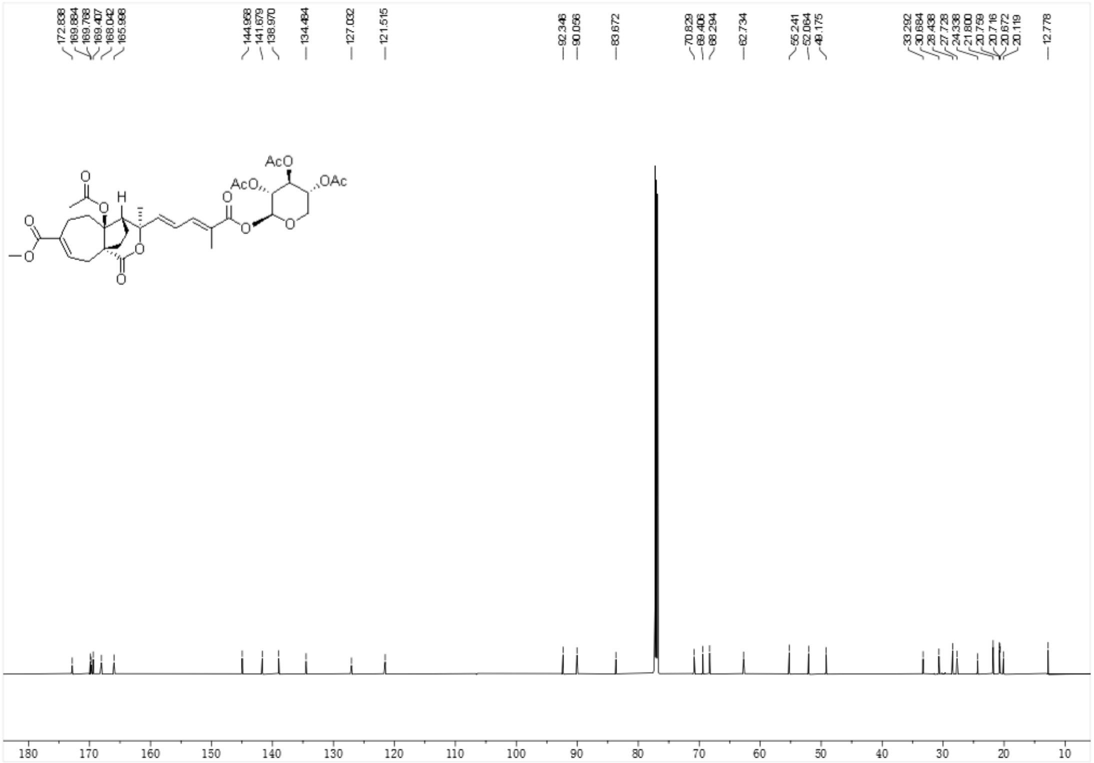

Figure S25. ^{13}C NMR spectrum of **17** (700 MHz, $CDCl_3$)

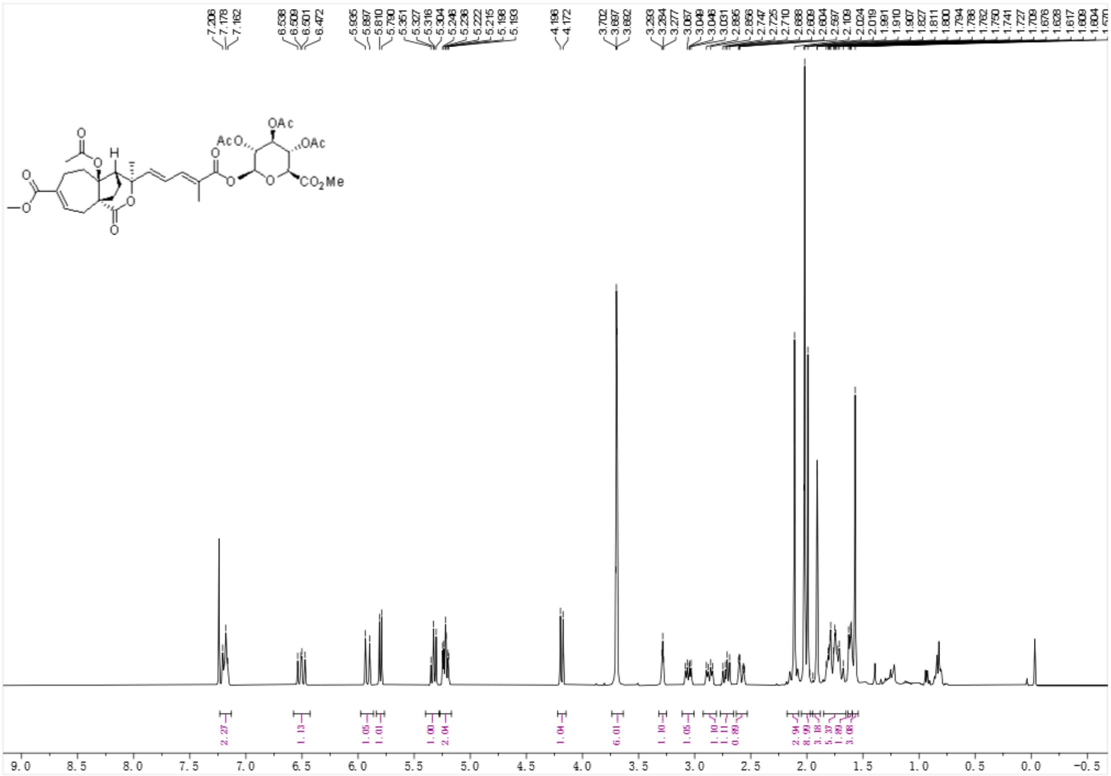

Figure S26. ^{1}H NMR spectrum of **18** (400 MHz, $CDCl_3$)

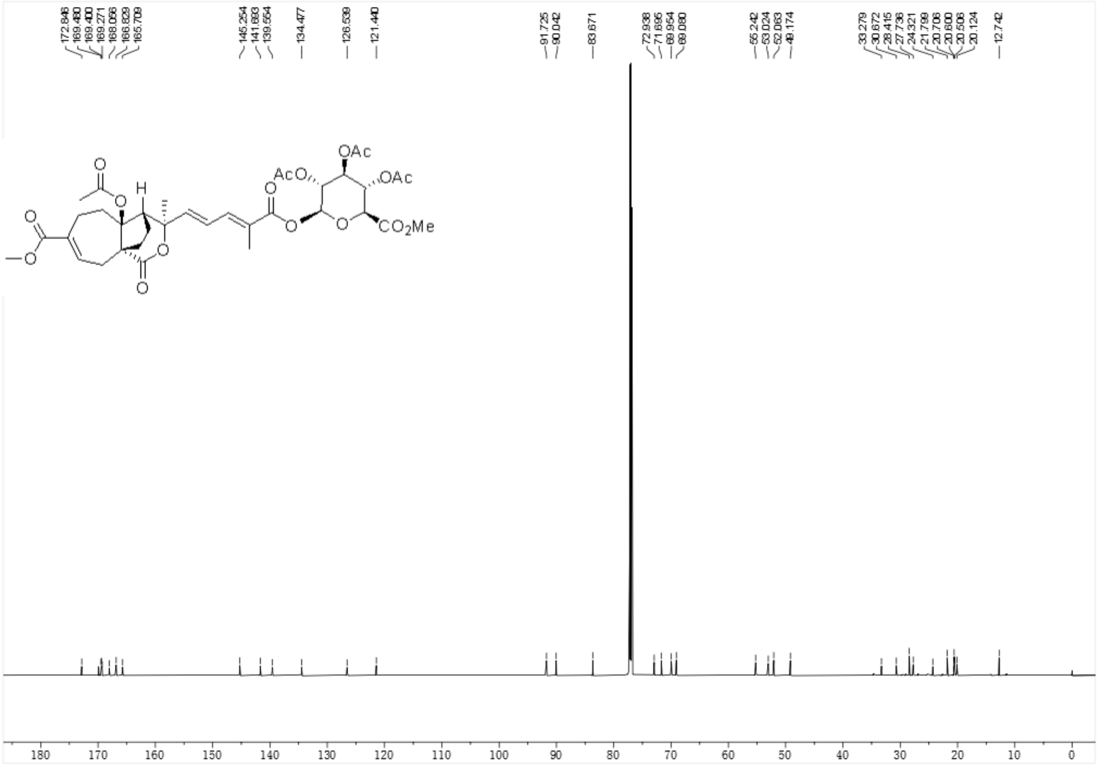

Figure S27. ^{13}C NMR spectrum of **18** (700 MHz, $CDCl_3$)

Table S1. RT-PCR primer sequences

Primer[a]	Sequence (5' to 3' direction)
**mARG1-F**	CATATCTGCCAAAGACATCGTG
**mARG1-R**	GACATCAAAGCTCAGGTGAATC
**mNOS2-F**	AATCTTGGAGCGAGTTGTGG
**mNOS2-R**	CAGGAAGTAGGTGAGGGCTTG
**mCD206-F**	CCTATGAAAATTGGGCTTACGG
**mCD206-R**	CTGACAAATCCAGTTGTTGAGG
**mMHC II-F**	CTGTCTGGATGCTTCCTGAGTTT
**mMHC II-R**	CAGCTATGTTTTGCAGTCCACC
**mRPL32-F**	GCTGGAGGTGCTGCTGATGTG
**mRPL32-R**	GGCGTTGGGATTGGTGACTCTG

[a]F = Forward Primer, R = Reverse Primer.
